# Effect of Boron and Vanadium Addition on Friction-Wear Properties of the Coating AlCrN for Special Applications

**DOI:** 10.3390/ma14164651

**Published:** 2021-08-18

**Authors:** Huu Chien Nguyen, Zdeněk Joska, Zdeněk Pokorný, Zbyněk Studený, Josef Sedlák, Josef Majerík, Emil Svoboda, David Dobrocký, Jiří Procházka, Quang Dung Tran

**Affiliations:** 1 Department of Mechanical Engineering, Faculty of Military Technology, University of Defence, 612 00 Brno, Czech Republic; huuchien.nguyen@unob.cz (H.C.N.); zdenek.pokorny@unob.cz (Z.P.); zbynek.studeny@unob.cz (Z.S.); emil.svoboda@unob.cz (E.S.); david.dobrocky@unob.cz (D.D.); jiri.prochazka@unob.cz (J.P.); 2 Department of Industrial Engineering and Information Systems, Faculty of Management and Economics, Tomas Bata University in Zlin, 760 01 Zlin, Czech Republic; sedlak@utb.cz; 3 Faculty of Special Technology, Alexander Dubcek University of Trencin, 91101 Trencin, Slovakia; jozef.majerik@tnuni.sk; 4 Faculty of Mechanical Engineering, Le Quy Don Technical University, Hanoi 100000, Vietnam; tranquangdung79@lqdtu.edu.vn

**Keywords:** H13, HS6-5-2, AlCrBN, AlCrVN, nanohardness, friction, wear resistance, adhesion

## Abstract

Cutting tools have long been coated with an AlCrN hard coating system that has good mechanical and tribological qualities. Boron (B) and vanadium (V) additions to AlCrN coatings were studied for their mechanical and tribological properties. Cathodic multi-arc evaporation was used to successfully manufacture the AlCrBN and AlCrVN coatings. These multicomponent coatings were applied to the untreated and plasma-nitrided surfaces of HS6-5-2 and H13 steels, respectively. Nanoindentation and Vickers micro-hardness tests were used to assess the mechanical properties of the materials. Ball-on-flat wear tests with WC-Co balls as counterparts were used to assess the friction-wear capabilities. Nanoindentation tests demonstrated that AlCrBN coating has a higher hardness (HIT 40.9 GPa) than AlCrVN coating (39.3 GPa). Steels’ wear resistance was significantly increased by a hybrid treatment that included plasma nitriding and hard coatings. The wear volume was 3% better for the AlCrBN coating than for the AlCrVN coating on H13 nitrided steel, decreasing by 89% compared to the untreated material. For HS6-5-2 steel, the wear volume was almost the same for both coatings but decreased by 77% compared to the untreated material. Boron addition significantly improved the mechanical, tribological, and adhesive capabilities of the AlCrN coating.

## 1. Introduction

Today, great emphasis is paid to corporate environmental policies [1], with innovative usage of plasma nitriding and PVD coatings replacing traditional, less ecologically friendly techniques. Due to their high hardness, outstanding wear resistance, superior corrosion, oxidation resistance, and good thermal stability, AlCrN thin coatings have been more important in industry over the last few decades [2,3]. At high temperatures, AlCrN coatings exhibit exceptional oxidation resistance due to the production of protective mixed protective oxides Al_2_O_3_, Cr_2_O_3_ [3,4]. As a result, they are commonly employed in the automobile sector as abrasion-resistant layers (e.g., valves, tappets, and camshafts) or as protective coatings for forming and machining tools [4,5]. At room temperature, AlCrN coatings had a high friction coefficient of 0.7, which climbed to 1.0 at temperatures above 500 °C, a temperature range often encountered in cutting operations [6]. The addition of alloying elements such as Si, B, and V [2,7,8] could be one way to significantly improve the film characteristics. The components Si and B have been shown to enhance the creation of a nanocomposite structure in AlCrN coatings, improving hardness, toughness, and/or wear resistance [9]. Due to the creation of a V_2_O_5_-Magnéli oxide phase at high temperatures, the inclusion of V proved to be effective in reducing friction, especially at high temperatures [2].

Many studies have looked into AlCrVN coatings to increase the friction-wear properties of materials [2,10,11]. Because of the formation of V_2_O_5_ oxide during tribological testing at extreme temperatures, the AlCrVN coatings have a low friction coefficient (0.2–0.3) and good wear resistance at high temperatures (700 °C). The AlCrVN coating greatly increased cutting tool performance while also boosting anti-wear ability due to the high hardness and produced lubricating V_2_O_5_ coatings [11,12]. The findings indicate that AlCrVN hard coatings have the potential to improve the wear resistance of cutting tools, forming, mechanical, and weapon parts [10,11].

Sato et al. [7] and Nose et al. [13] reported that boron addition improved the mechanical properties of AlCrN coatings by combining the effects of solid solution hardening, grain size refinement (Hall–Petch hardening), and formation of nanocomposite structure, where a-BNx tissue phase embedded the AlCrN crystallites. The nanocomposite structure of AlCrBN coatings, in which nano-sized fcc AlCrN grains are surrounded by a thin BNx tissue phase, greatly increased the hardness of the AlCrN coatings while reducing compressive residual stress [14]. At room temperature and at the evaluated temperature (700 °C), AlCrBN coatings have recently been shown to have superhardness and high wear resistance [9]. Boron addition reduced the grain size of the AlCrN coatings from 40 nm to around 10 nm, and the internal stress of the coating system was lowered by more than 50% [15]. In comparison to AlCrN and AlCrTiN coatings, the AlCrBN coating resists gear hobbing against crater wear the best [15].

It has been observed that a hybrid surface treatment consisting of plasma nitriding and hard coatings significantly improved the mechanical and friction-wear properties of materials, as well as the coating adhesion strength [16,17,18]. Plasma nitriding, which generates a harder barrier between the soft substrate and the hard coating [19], plays a significant role in this hybrid surface treatment [16]. The nitrided hardened layer considerably reduces the plastic deformation [20] that happens underneath the coating. As a result, the coatings’ durability and adhesion strength will be enhanced [17,21].

Although the microstructure, hardness, and wear resistance of AlCrBN and AlCrVN coatings have been extensively studied, the influence of surface treatments such as plasma nitriding and AlCrBN, AlCrVN coatings on the friction-wear properties of steel has still to be researched.

The purpose of this investigation was to see how a hybrid treatment consisting of plasma nitriding and AlCrVN, AlCrBN coating affected the friction-wear properties of H13 tool steel and HS6-5-2 high-speed tool steel. H13 hot-work steel is commonly used in both hot and cold work tooling. Cutting tools are commonly made of HS6-5-2 high-speed steel. Under the same conditions, AlCrVN and AlCrBN coatings were applied to the surfaces of both un-nitrided (only-coating) and nitrided (duplex coating) materials. The mechanical properties and wear resistance of the AlCrBN and AlCrVN multicomponent coatings were investigated and compared. The results from the un-nitrided materials, plasma-nitrided materials, and only-coated materials were compared to the results from the hybrid-surface-treated materials.

## 2. Materials and Methods

### 2.1. Materials

H13 steel and HS6-5-2 high-strength steel were chosen for use in the experiment. The chemical composition of the steel was assessed five times by Q4 TASMAN spark emission equipment (Bruker, Karlsruhe, Germany), with the averages used as data. The chemical compositions of H13 and HS6-5-2 steels are shown in Table 1 and Table 2, respectively.

Steel H13 samples have a diameter of 65 mm and a thickness of 6 mm, while steel HS6-5-2 samples have a diameter of 20 mm and a thickness of 3 mm. After heat treatment, all experimental samples were supplied. The heat-treated samples had a surface hardness of 52 HRC for steel H13 and 64 HRC for HS6-5-2 steel.

Surface roughness Ra of 0.11 µm for steel H13 and 0.04 µm for steel HS6-5-2 was obtained by grinding the samples using a Struers LaboSystem (Struers, Copenhagen, Denmark) grinder. Silicon carbide grinding paper grits 120, 220, 400, 600, and 800 were utilized for grinding. The cross sections of the specimens were polished to mirror surfaces using Leco CAMEO Disc Platinum (Leco Corporation, St. Joseph, MI, USA) 1, 2, 3, 4 and diamond polishing paste with grain size 1 µm by Leco PX-500 Grinder/Polisher (Leco Corporation, St. Joseph, MI, USA) for microstructure characterization. After that, a picric acid solution (1 g picric acid, 5 mL HCl acid in 100 mL ethanol) was used to etch the polished cross sections of steel HS6-5-2, and a 2 percent Nital etchant (a solution of ethanol and nitric acid) was used to etch the polished cross sections of steel H13. Plasma nitriding was carried out using PN60/60 RÜBIG equipment (Rubig GmBH, Wels, Austria) at 470 °C for 4 h with a mixture gas of 3H_2_:1N_2_ at 280 Pa. UN and PN materials stand for un-nitrided and plasma-nitrided materials, respectively. The cathodic arc PVD process was utilized to coat AlCrBN and AlCrVN. The Pi411 equipment was used to coat AlCrBN and AlCrVN coatings on both un-nitrided and nitrided materials in the company LISS. Un-nitrided AlCrBN-coated and AlCrVN-coated materials are referred to as AlCrBN material and AlCrVN material, respectively. The PN/AlCrBN and PN/AlCrVN materials (respectively duplex-coated materials) are AlCrBN-coated and AlCrVN-coated nitrided materials.

### 2.2. Experiment Procedures

The surface roughness of materials was measured using absolute inductive position sensor by Talysurf CLI 1000 stylus profilometer (Taylor Hobson Ltd., Leicester, England). For evaluation of the surface, the parameter Ra was used—the arithmetic average of the absolute values of the profile heights over the evaluation length. The microstructures were observed on the cross sections of the specimens by Olympus DSX 500i opto-digital microscope(Olympus, Tokyo, Japan). The features of the coatings were observed, and the coatings thickness was measured on the cross sections of the coated specimens by a Tescan Mira 4 scanning electron microscope (Tescan, Brno, Czech Republic).

The surface hardness and Young’s modulus of the UN and PN materials were measured using the instrumented indentation test by the Zwick ZHU 2.5 hardness tester (Zwick, Brno, Czech Republic). For the instrumented indentation test, the test force of 49.81 N and dwell time of 12 s were used. These measurements were performed ten times and the averages were used as the data. The microhardness profiles of the nitrided layers were obtained in the range from the surfaces to 0.5 mm depth on the polished cross sections. The microhardness was measured using the Vickers method on the Leco AMH55 hardness tester (Leco Corporation, St. Joseph, MI, USA). For the microhardness measurement the test force of 0.981 N and dwell time of 12 s were used. The microhardness measurement was carried out three times at each depth and their average values were used as the experimental values. The depth of nitrided layer was evaluated base on the results of measurement microhardness profile according to ISO-18203 [22]. The nanohardness and Young’s modulus of the AlCrBN and AlCrVN coatings were measured 10 times using a nano-indenter, and their averages were used as the data.

The adhesion of the coatings was evaluated by the scratch test using the Universal Mechanical Tester 3 (UMT-3) and by the Rockwell indentation test using an Indentec HRC 8150 LK hardness tester (Zwick/Roell GmbH, Ulm, Germany). For these tests, the Rockwell diamond indenter with a rounded tip in radius of 200 µm was used. In the scratch test, the sliding speed was 0.17 mm/s, and the moving distance was 10 mm. The normal load was linearly increased from 5 to 150 N and 200 N for only-coated and duplex-coated materials, respectively. The critical loads were determined using the signal of acoustic emission and friction coefficient. For the Rockwell indentation test the test force was 1471 N and dwell time was 12 s. The tests were performed three times for all coatings. The friction-wear qualities of the materials were assessed using the linearly reciprocating ball-on-flat method. Friction-wear experiments were conducted at room temperature in air using a UMT-3 tester (Bruker, Karlsruhe, Germany) with no lubrication. The opposing substance was tungsten carbide balls with a diameter of 6.35 mm. The test load was set to 10 N. Table 3 depicts the test circumstances.

The features of wear tracks were evaluated using an Olympus DSX 500i optical microscope (Olympus, Tokyo, Japan) after the friction-wear testing. The wear track profiles were collected using an absolute inductive position sensor and a Talysurf CLI 1000 stylus profilometer (Taylor Hobson Ltd., Leicester, England). At eleven points along the wear track, profile measurements were taken. The mean cross-sectional profile was calculated from the measured profiles, and Talysurf Platinum software (v5, Taylor Hobson Ltd., Leicester, England) was used to calculate the area and depth of worn track cross sections. From the cross-sectional area and stroke length, the total volume of material lost during sliding (wear volume) was estimated [23].

## 3. Results

### 3.1. Surface Roughness

**Figure 1:**
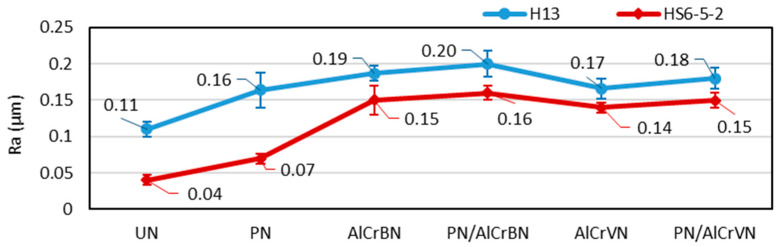
Surface roughness of materials.

The surface roughness (Ra) of the studied materials is depicted in Figure 1.

In comparison with UN materials, the surface roughness Ra value of the PN material was 45% greater for H13 steel and 72% higher for HS6-5-2 steel. For steel H13, the surface roughness values were 50% to 70% higher than the UN material. Such deterioration is caused by a dedusting process, when the nitride cations bombard a material surface and subsequently atoms of various elements, being on a material surface are shot out [24]. The surface roughness of the coatings on steel HS6-5-2 was 245 percent to 295 percent higher than the UN material. Higher values of the surface roughness of the coating’s parameters Ra and Rz have a detrimental impact on the coated part’s friction, wear, and service life. Methods of surface treatment of coatings can be applied after coating deposition to minimize the values of the parameters Ra of surface roughness. Surface treatment of the coating with wet sandblasting and lap technologies reduces the value of the Ra roughness parameters of the coated surface [25].

### 3.2. Microstructures

**Figure 2:**
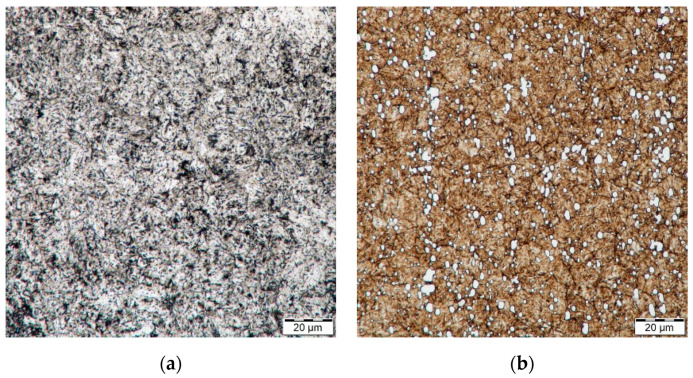
Microstructure of steel (**a**) H13; (**b**) HS6-5-2 with magnification 1000×.

**Figure 3:**
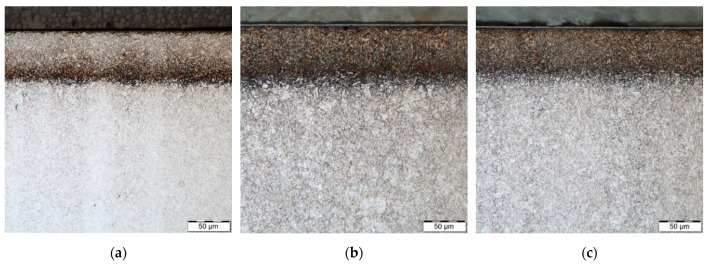
Microstructure optically observed with magnification 1000× on the steel H13 samples cross sections: (**a**) PN material; (**b**) PN/AlCrBN material; (**c**) PN/AlCrVN material.

**Figure 4:**
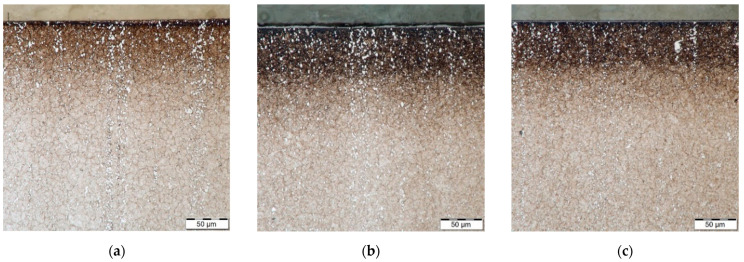
Microstructure optically observed on the steel HS6-5-2 samples cross sections: (**a**) PN material; (**b**) PN/AlCrBN material; (**c**) PN/AlCrVN material.

**Figure 5:**
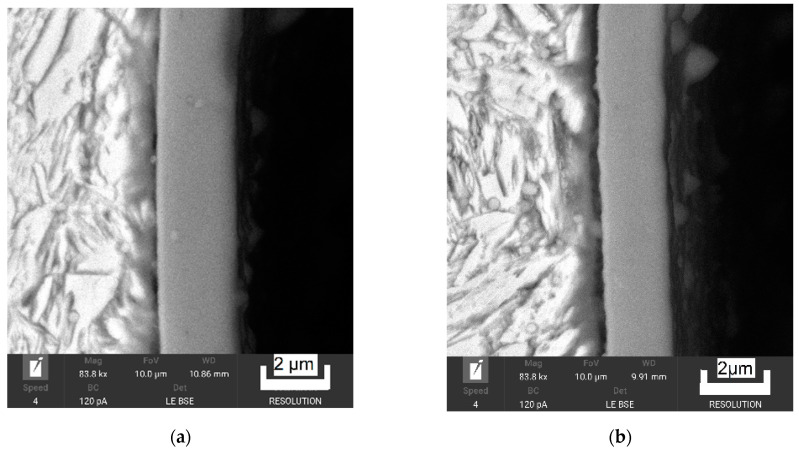

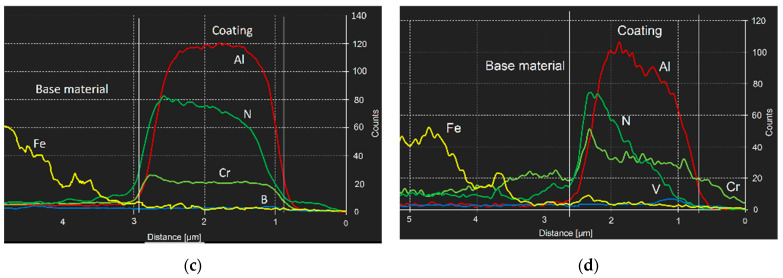
Features of the coatings on nitrided steel H13: (**a**) AlCrBN; (**b**) AlCrVN; (**c**) EDS line scan of AlCrBN coating; (**d**) EDS line of AlCrVN coating.

The microstructure of H13 and HS6-5-2 steels is shown in Figure 2. Figure 3, Figure 4 and Figure 5 demonstrate the microstructures found along the surfaces of PN, PN/AlCrBN, and PN/AlCrVN materials on cross sections.

Steel H13 is a pearlitic steel with secondary carbides that is subledeburitic. The microstructure of H13 steel is tempered martensite following heat treatment, as shown in Figure 2a. The ledeburitic steel HS6-5-2 has a pearlitic structure. The martensite microstructure was detected in heated steel HS6-5-2, as shown in Figure 2b. The material HS6-5-2 has a fine-grained structure with carbides that are evenly distributed.

Figure 3a and Figure 4a show the diffusion layers of the PN materials; nevertheless, no compound layer was generated. The duplex coatings, which include a thin coating and a thick nitrided layer, were formed on the cross sections of the duplex-coated materials (Figure 3b,c and Figure 4b,c). The internal microstructures of PN materials, duplex-coated materials, and UN materials were identical, as illustrated in Figure 2, Figure 3 and Figure 4. Due to their low conducting temperature, plasma nitriding and AlCrBN, AlCrVN coatings have little effect on the interior microstructures of steels.

Figure 5 depicts the characteristics of the AlCrBN and AlCrVN coatings as observed by SEM on cross sections.

On AlCrBN-coated materials, single coatings with a thickness of 2.0 µm were generated, as illustrated in Figure 5a. On the cross sections of AlCrVN-coated materials, a single coating of AlCrVN with a thickness of 1.8 µm was detected (Figure 5b).

### 3.3. Hardness

In Table 4, the surface hardness of UN and PN materials is compared.

**Figure 6:**
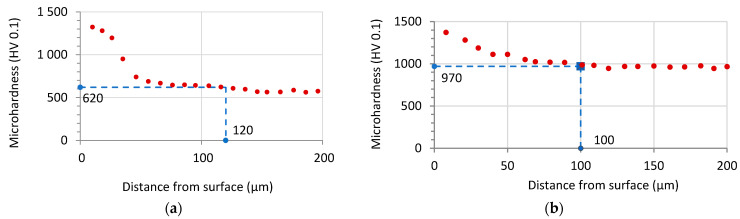
Microhardness profiles of the nitrided samples: (**a**) steel H13; (**b**) steel HS6-5-2.

Plasma nitriding increased surface hardness by 123 percent for steel H13 and by 53 percent for steel HS6-5-2, as shown in Table 4. The microhardness profiles on the cross sections of PN materials are shown in Figure 6. The hardened layers were created by the plasma nitriding method, as shown in Figure 6. Microhardness profiles according to ISO-18203 [22] were used to determine the depth of the nitrided diffusion layers. The gradual decrease in hardness from the surface towards the core is due to the diffusion mechanism of the penetration of nitrogen atoms into the base material.

Table 5 displays the maximum and minimum microhardness values, as well as the depth of nitrided layers.

Great variations in the mechanical characteristics of the two materials might lead to probable failure zones due to elastic and plastic incompatibility in only-coated systems, where a hard ceramic coating is put over a soft substrate material [16,21].

The nitrided layer was designed to improve the substrate load-bearing capacity in duplex-coated materials by providing a progressive transition in mechanical characteristics between the substrate and the hard ceramic coating [21,25]. The creation of the nitrided layer considerably increased the adhesive strength and tribological properties of ceramic coatings [17]. The ratio H/E*, where E* = E/(1—v^2^) and v is the Poisson’s ratio, was commonly used to assess resistance to elastic strain to failure in the surface-contact mode, which is obviously significant for avoiding wear [26,27,28]. The resistance to plastic deformation in a surface contact system is measured using H^3^/E*^2^ values [13,26,27,29].

Indentation hardness of UN and PN materials, nanohardness of AlCrBN and AlCrVN coatings evaluated by indentation test, and H/E* and H^3^/E*^2^ ratios of tested materials are shown in Table 6.

The highest H/E* and H^3^/E*^2^ ratios of 0.090 and 0.3302 GPa, respectively, were observed for the coating AlCrBN, as shown in Table 6. The values associated with the AlCrVN coating were slightly lower. The ceramic coatings had substantially higher values than the PN materials.

For steel HS6-5-2, the value of H/E*, H^3^/E*^2^ corresponding to the nitrided layer was about half and four times that of un-nitrided material. For steel H13, the nitride material enhanced the value of H/E* and H^3^/E*^2^ by nearly twice and ten times, respectively, as compared to un-nitrided material.

### 3.4. Adhesion Strength

One of the most essential properties of ceramic coatings is adhesion strength. It determined the service life of coating materials in a direct manner [30]. The Rockwell indentation and scratch test were frequently used to evaluate the adhesion strength of ceramic coatings to substrates.

**Figure 7:**
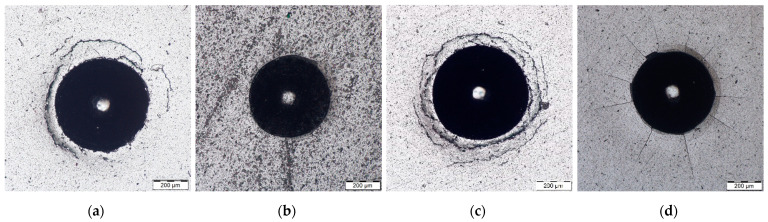
The optical micrograph of Rockwell indentation of the coatings on substrate steel H13 with magnification 250×: (**a**) AlCrBN; (**b**) PN/AlCrBN; (**c**) AlCrVN; (**d**) PN/AlCrVN.

**Figure 8:**
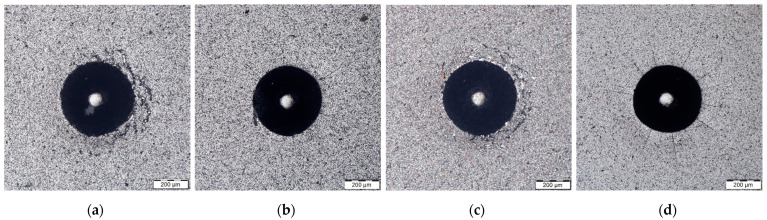
The optical micrograph of Rockwell indentation of the coatings on substrate steel HS6-5-2 with magnification 250×: (**a**) AlCrBN; (**b**) PN/AlCrBN; (**c**) AlCrVN; (**d**) PN/AlCrVN.

The optical micrographs of Rockwell indentation of AlCrBN coatings and PN/AlCrBN duplex coatings on substrate steel H13 and steel HS6-5-2, respectively, are shown in Figure 7 and Figure 8.

The substrates un-nitrided materials of indentation were extruded to the edge and certain bulges were generated, as shown in Figure 7a,c and Figure 8a,c. Around the margin of the indentation, circular cracks through coatings and tiny spallation of coatings developed. The adhesion strength of AlCrBN only-coating and AlCrVN only-coating generated on substrate steel H13 and steel HS6-52 can be categorized as HF3 and HF2, respectively, according to the Rockwell indentation test VDI 3198 standard [31].

**Figure 9:**
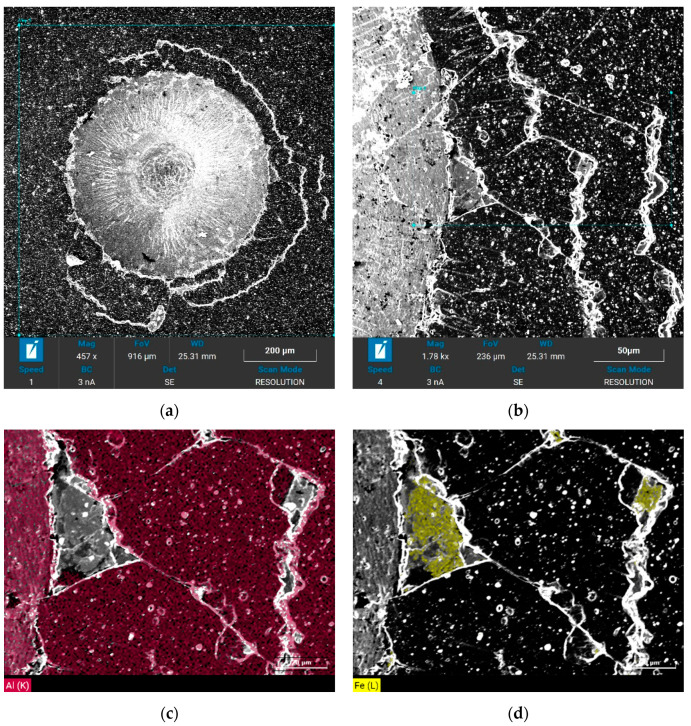
EDS analysis of cracks and the peeled AlCrVN coating: (**a**) surface feature of the indentation; (**b**) surface feature of the cracks and spallation; (**c**) distributions of Al; (**d**) distributions of Fe.

Because the coatings at the indentation edge rose during the loading process producing increased transverse shear stress and longitudinal tensile stress, and the AlCrBN and AlCrVN coatings exhibited high hardness and great brittleness, fractures formed under the compressive stress [32]. In comparison with substrates HS6-5-2, substrates H13 had larger indentation bulges due to their reduced hardness. The adhesion strength of the AlCrBN and AlCrVN coatings created on un-nitrided substrates steel H13 and steel HS6-52 was similar to HF2 when compared to the quality level of bonding strength according to Rockwell indentation test VDI 3198 standards. Figure 9 shows an EDS analysis of the neighborhood of indentation after the indentation test. An analysis of the presence of the two main elements that occur in the coating and the base material, Al and Fe, was performed. The occurrence of Fe was measured in the area of the peeled off part of the coating, which means that the peeling took place in the whole volume of the coating and not in its layers.

The diameter of the indentations (respectively plastic defamation) was larger in the duplex-coated materials than in the only-coated materials. It was discovered that the surface sink-in around the indentations had occurred. Figure 7b,d and Figure 8b,d show a few cracks around the margin of the indentation on the surface of the duplex-coated materials. The adhesion strength of AlCrBN and AlCrVN coatings produced on nitrided steel H13 and steel HS6-52 substrates was comparable to that of HF1. As can be seen in Figure 7 and Figure 8, the AlCrVN coatings had a greater number of cracks and longer cracks on their surface than the AlCrBN coatings. According to the findings, AlCrBN coatings have a higher toughness than AlCrVN coatings.

**Figure 10:**
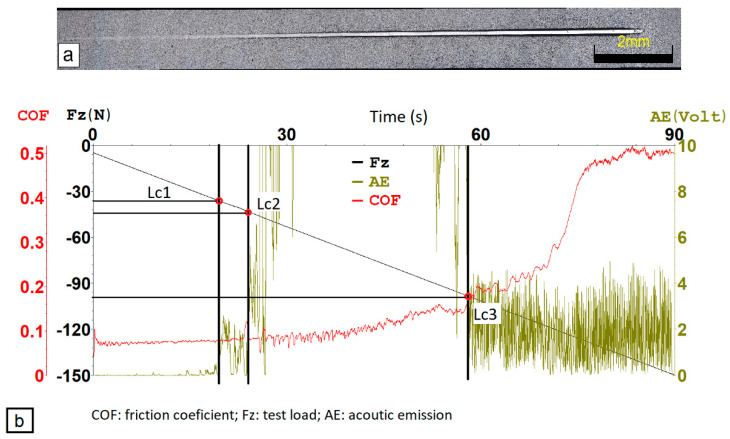
Critical loads of the coating AlCrBN on un-nitrided substrate of steel HS6-5-2 measured by scratch test: (**a**) optical micrographs of scratch scar; (**b**) relationship between the COF, AE, Fz and time test.

Figure 9a,b show SEM picture of crater after indentation test with EDS analyses of spallation. By analyzing the elements Fe and Al in Figure 9c,d, it was found that the coating peeled off in its entire thickness. Figure 10 depicts the critical loads of AlCrBN coating on un-nitrided substrate steel HS6-5-2, scratch optical micrographs, and the link between friction coefficient, acoustic emission, test load, and time test assessed by scratch.

The critical load is defined as the least load at which a discernible failure occurs in a scratch test with progressive load [33]. The scratch test has three stages that correspond to three critical loads [30,32,34,35,36]. Each failure event, such as coating cracking and delamination, causes acoustic emission and a change in the coefficient of friction. The first AE peak and friction oscillation correspond to the first critical load Lc1, which is typically attributed to the first crack event. The delamination of coatings with substrate exposure determines the second critical load Lc2, which is usually connected with the adhesion strength between the coating and the substrate. The development of a quick increase in AE and random changes in the friction coefficient signal the start of delamination. The third critical load Lc3, which corresponds to the complete removal of a coating from the scratch groove, can be calculated using a microscopic study of the scratch tracks and a quick increase in coefficient friction. The second critical load, Lc2, is often seen as a symptom of coating adhesion failure [30].

**Figure 11:**
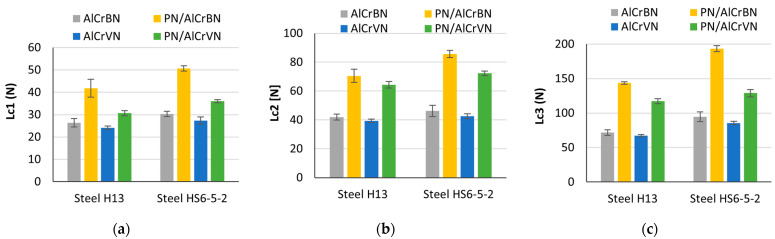
Critical loads of the AlCrBN and AlCrVN coatings formed on different substrates: (**a**) first critical load Lc1; (**b**) second critical load Lc2; (**c**) third critical load Lc3.

The critical loads of AlCrBN and AlCrVN coatings produced on various substrates are shown in Figure 11.

The AlCrBN nanocomposite coatings had a high hardness, but they had a lower compressive residual stress [14] and a lower internal stress of the coating system by >50% [15]. This improves the hardness [7,14,37] and adhesive strength of the AlCrBN coatings [7,27]. The film’s resistance to cracking improves as the H^3^/E*^2^ ratio rises [27]. As demonstrated in Table 6, the ratio H^3^/E*^2^ of the AlCrBN coating was higher than that of the AlCrVN coating. As a result, the critical loads of the AlCrBN coating for substrates were higher than those of the AlCrVN coating, as shown in Figure 11. 

Coatings formed on steel HS6-5-2 substrates had higher adherence than steel H13 substrates, and coatings formed on nitrided substrates had substantially higher adhesion than un-nitrided substrates. The findings indicated that the nitrided layer improved the substrate load-bearing capability by allowing for a progressive change in mechanical characteristics between the substrate and the hard coating, resulting in a significant increase in the adhesion strength of the AlCrBN and AlCrVN coatings.

### 3.5. Friction-Wear Properties

**Figure 12:**
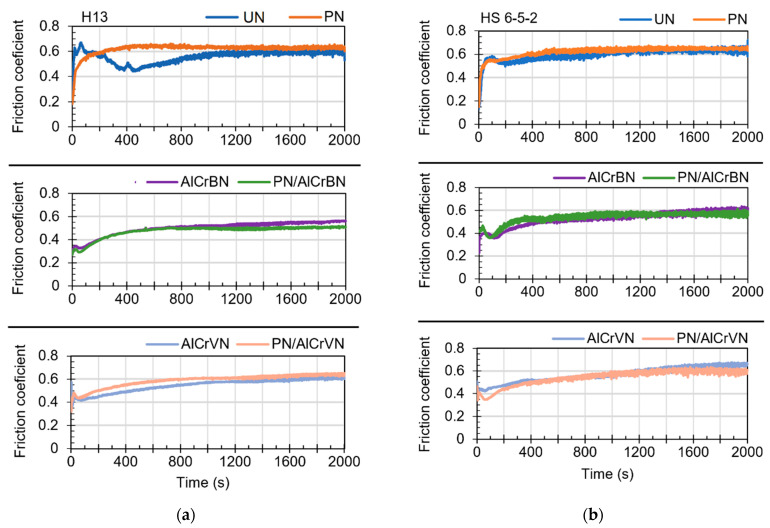
Relationship between the test time and friction coefficients of the tested materials (**a**) Steel H13; (**b**) Steel HS6-5-2.

**Figure 13:**
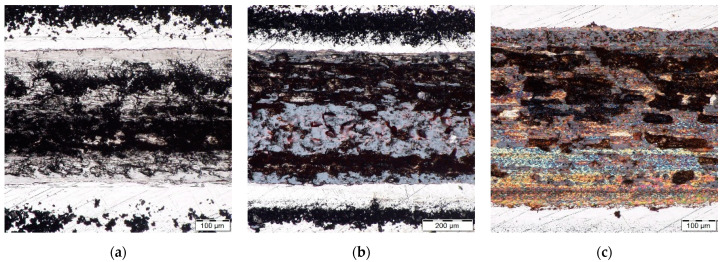
Morphology of the wear tracks on UN material of steel H13 after finishing addition tribological tests at time of: (**a**) 200 s; (**b**) 400 s; (**c**) 1000 s.

The relationship between the test period and the friction coefficients of all the investigated materials is depicted in Figure 12. Figure 12 shows that the UN steel H13 material has an unstable friction coefficient ranging from 0.42 to 0.66. To investigate the cause of the decrease in friction coefficient, three further wear tests were performed on UN material of steel H13. The first tribological test took 200 s to complete, corresponding to a friction coefficient of roughly 0.6. The second addition tribological test was completed in 400 s, equating to a friction coefficient of 0.42. The third tribological test took 1000 s to complete, equal to a friction coefficient of roughly 0.6. Figure 13 depicts the morphology of the wear tracks after additional tribological testing have been completed. The untreated sample has a morphology indicative of ploughing and oxidation, as seen in Figure 13. This is due to the fact that the H13 steel substrate has a lower hardness than the corresponding material (WC-Co 6 percent). The friction coefficient of two sliding contact surfaces is affected by the deposition of wear debris layers on wear track surfaces [38,39]. For the first addition wear test, the wear debris and sintered wear debris layers generated on the side of the wear track were detected (Figure 13a). Figure 13b shows how sintered wear debris layers cover practically the entire surface of the wear track, increasing the friction coefficient from roughly 0.6 to 0.42.

Figure 13c depicts the dispersed dispersion of thin wear debris layers. The worn surface was heavily oxidized. The removal of the wear debris layers from the wear track surface can be caused by a change in the surface of the counterpart material (ball). The ball was worn out during the test, and the surface of the balls that came into touch with the samples became flat. As a result of the ball ploughing, the wear debris layers are forced away from the wear track surface.

The tribological tests included two stages, referred to as the running-in stage and the stable stage, respectively, as shown in Figure 11. Except for un-nitrided steel H13, which started at a test time of 1000 s, the stable stages of tested materials began at around 400 s. For each test, the measured value of the friction coefficient is derived as the average of the stable region of the friction coefficient curve.

Table 7 shows the results of using three measured friction coefficients for tested materials to calculate the final values.

**Figure 14:**
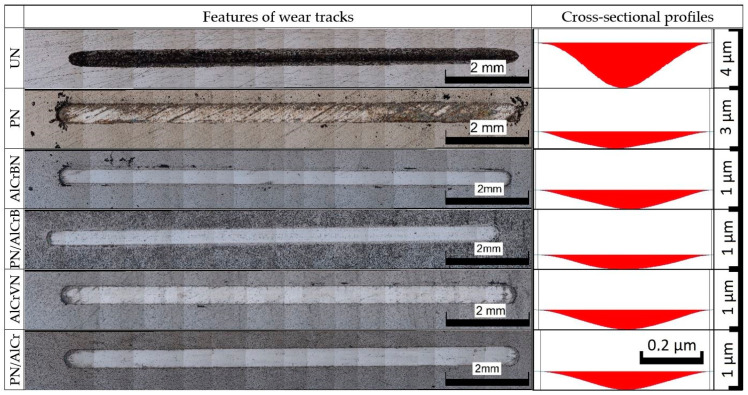
Features and cross-sectional of wear tracks of tested materials steel H13.

**Figure 15:**
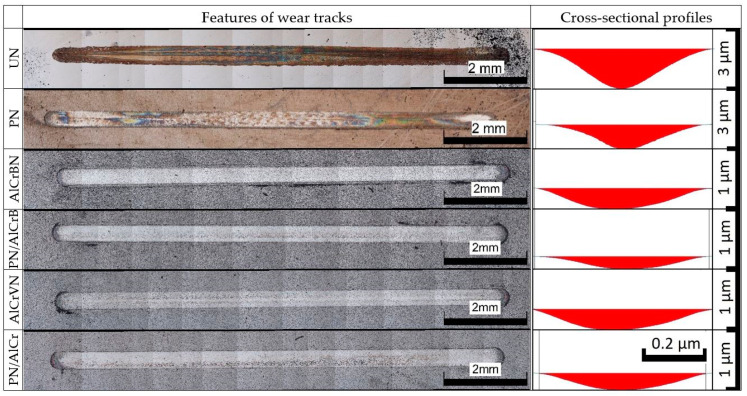
Features and cross-sectional of wear tracks of tested materials steel HS6-5-2.

The characteristics and cross-sectional profiles of the wear tracks of the investigated samples steel H13 and HS6-5-2 are shown in Figure 14 and Figure 15.

The PN materials had the highest friction coefficient, as seen in Table 7 (0.63 and 0.64 for steel H13 and HS6-5-2, respectively). The friction coefficient values for the UN materials and AlCrVN coated materials varied between 0.6 and 0.7. The AlCrBN coated materials had the lowest friction coefficient, which was around 0.53.

Surface contact area, surface shear strength, surface hardness, and surface roughness all affect the friction coefficient [17,40]. The wear debris layers that can accumulate during a tribological test can also affect the friction coefficient [38,39]. The friction coefficient is influenced by the material’s microstructure [41,42,43]. Even if the hardness is raised, the wear resistance is enhanced [17]. Friction coefficients do not always decrease.

Coating failures did not occur, and no substrate exposure was discovered on the wear tracks of the coated materials, as illustrated in Figure 14 and Figure 15.

**Figure 16:**
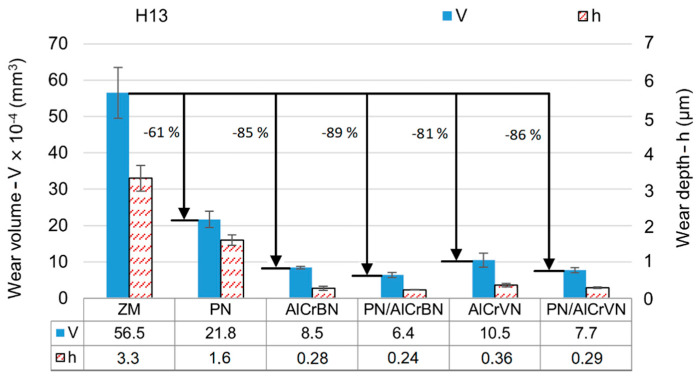
Wear volumes and wear depth of the tested materials of steel H13.

The wear volumes and depths of the tested materials of steel H13 and HS6-5-2 steel are shown in Figure 15 and Figure 16, respectively.

**Figure 17:**
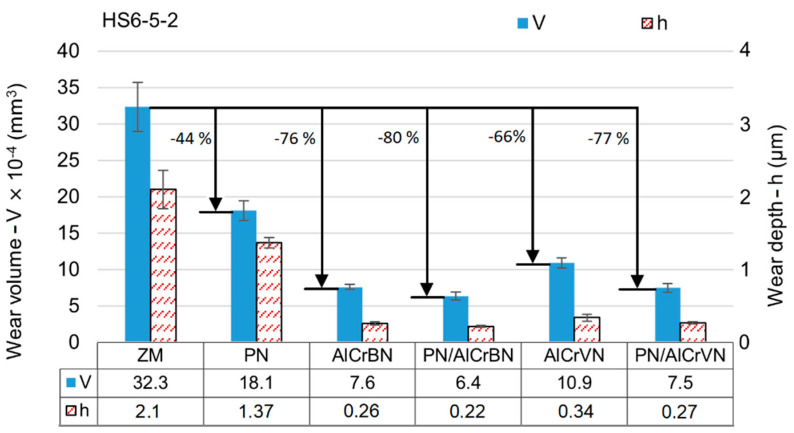
Wear volumes and wear depth of the tested materials of steel HS6-5-2.

As shown in Figure 16 and Figure 17, the wear depths of all the coated materials were lower than the coating thickness. The wear resistance of surface materials is generally related to the surface hardness, and then to the H/E* and H^3^/E*^2^ ratios. The hardness and H/E* and H^3^/E*^2^ ratios of the treated materials were higher than those of the untreated materials, as shown in Table 4, Table 5 and Table 6. As a result, for both steel H13 and HS6-5-2, the wear volumes of all treated materials (PN, only-coated, and duplex-coated) were lower than the steel substrate. PN materials exhibited 61 percent and 44 percent lower wear volumes for steel H13 and HS6-5-2, respectively, when compared to untreated materials. Wear volume reductions ranged from 81 percent to 89 percent for coated steel H13 materials and 66 percent to 80 percent for coated steel HS6-5-2 materials.

The development of nitrided layers reduced plastic deformation caused by the coatings and raised the H/E* and H^3^/E*^2^ ratios of the duplex-coated materials. In all situations, duplex-coated materials had lower wear volume values by 16% to 31% as compared to only-coated materials, according to the results of wear volume measurement. The AlCrBN coating displayed excellent hardness, low compressive residual stress, good toughness, and good wear resistance by combining the effects of solid solution hardening, grain size refinement (Hall–Petch hardening), and development of nanocomposite structure by boron addition [9,14,15]. The production of V_2_O_5_, which can operate as a liquid lubricant and reduce friction coefficients from 0.6–0.8 to 0.2–0.3 at high temperatures (700 °C), was one of the key benefits of the vanadium addition [11,44]. In fact, for steel H13 and HS6-5-2, the wear volume of the AlCrVN only-coated material was 24% and 43% higher than that of the AlCrBN only-coated material, respectively. For steel H13 and HS6-5-2, the PN/AlCrVN duplex-coated material had a 20 percent and 18 percent higher wear volume than the PN/AlCrBN duplex-coated material, respectively.

## 4. Conclusions

The purpose of this investigation was to see how surface roughness, mechanical characteristics, and friction-wear parameters of H13 and HS6-5-2 steel were affected by a hybrid treatment that included plasma nitriding and AlCrVN, AlCrBN ceramic coating. The effects of adding boron and vanadium to the AlCrN coating on mechanical, adhesion strength, and friction-wear properties were investigated.

The harder layers generated by plasma nitriding significantly increased the adherence of the AlCrBN and AlCrVN coatings. The AlCrBN coatings had greater adherence than the AlCrVN coatings.

H/E* and H^3^/E*^2^ ratios of 0.090 and 0.3302 GPa, respectively, were highest in the AlCrBN coating. The AlCrVN coating’s H/E* and H^3^/E*^2^ values, which were 0.089 and 0.3155, respectively, were a little lower than the AlCrBN coating’s. The coatings had substantially higher values than the PN materials. The value of H/E*, H^3^/E*^2^ corresponding to the nitrided layer in steel HS6-5-2 was roughly half and four times that of un-nitrided material, respectively.

Steels with a hybrid surface treatment consisting of nitriding and AlCrBN or AlCrVN coatings have considerably better friction-wear qualities than steels with simply coatings or nitrided layers.

The AlCrBN coating was harder, adhered better, had a lower friction coefficient, and was more resistant to wear than the AlCrVN coating. The results suggested that adding boron to the AlCrN system multicomponent coatings increased their mechanical and friction-wear qualities significantly.

## Figures and Tables

**Table 1:** H13 steel chemical composition (weight %).

	C	Mn	Si	Cr	Mo	V	P	S
ASTM A681	0.32–0.45	0.2–0.6	0.8–1.25	4.75–5.5	1.1–1.75	0.8–1.2	Max. 0.03	Max. 0.03
Measured	0.36	0.47	0.97	4.80	1.24	0.84	0.030	0.01

**Table 2:** HS6-5-2 steel chemical composition (weight %).

	C	Mn	Si	Cr	Mo	W	V	P	S
EN ISO 4957	0.8–0.88	Max. 0.4	Max. 0.45	3.8–4.5	4.7–5.2	5.9–6.7	1.7–2.1	Max. 0.03	Max. 0.03
Measured	0.82	0.35	0.23	4.50	5.35	1.94	7.00	0.028	0.010

**Table 3:** Conditions of the wear tests.

Stroke Length (mm)	Oscillating Frequency (Hz)	Test Duration (s)	Ambient Temperature (°C)	Relative Humidity (%)	Lubrication
10	3.5	2000	22 ± 0.5	40–60	None applied

**Table 4:** Surface hardness of the UN materials and PN materials.

Steel	Material	Hardness (HV 5)
H13	UN	502 ± 6
	PN	1120 ± 15
HS6-5-2	UN	874 ± 8
	PN	1335 ± 10

**Table 5:** Properties of the nitrided layers.

Steel	Maximal Microhardness Value (HV 0.1)	Limit Microhardness Value (HV 0.1)	Case Depth (µm)
H13	1325 ± 65	620	120 ± 12
HS6-5-2	1375 ± 52	970	100 ± 10

**Table 6:** Ratio H/E* and H^3^/E*^2^ of the tested materials.

Material-Steel	HIT (GPa)	E* (GPa)	H/E* × 10^−3^	H^3^/E*^2^ × 10^−3^ (GPa)
UN-H13	5.6 ± 0.4	230.8 ± 15	24 ± 3	3.3 ± 1.1
PN-H13	11.5 ± 0.6	224.2 ± 8	51 ± 5	30.3 ± 6.9
UN-HS6-5-2	9.5 ± 0.4	230.0 ± 10	41 ± 4	16.2 ± 3.5
PN-HS6-5-2	13.9 ± 0.5	237.4 ± 7	59 ± 4	47.7 ± 8
AlCrBN	40.9 ± 0.5	455.2 ± 9	90 ± 3	330.2 ± 25.2
AlCrVN	39.3 ± 0.5	438.3 ± 8	89 ± 3	315.5 ± 23.6

**Table 7:** Friction coefficient of tested materials.

Steel	UN	PN	AlCrBN	PN/AlCrBN	AlCrVN	PN/AlCrVN
H13	0.57 ± 0.05	0.63 ± 0.04	0.53 ± 0.02	0.50 ± 0.03	0.60 ± 0.03	0.62 ± 0.02
HS6-5-2	0.62 ± 0.04	0.64 ± 0.04	0.55 ± 0.02	0.56 ± 0.03	0.59 ± 0.03	0.57 ± 0.03

## Data Availability

Data are contained within the article.
